# Supplementary material for: A Global Screen for Assembly State Changes of the Mitotic Proteome by SEC-SWATH-MS
Source: Cell Syst. 2020 Feb 26;10(2):133–155.e6. doi: 10.1016/j.cels.2020.01.001 (PMC7042714; doi:10.1016/j.cels.2020.01.001)

Q96G28 | CFA36_HUMAN | CFAP36 CCDC104 UNQ163/PRO189
Monomer MW [kDa]: 39.447 Monomer expected elution fraction: 48

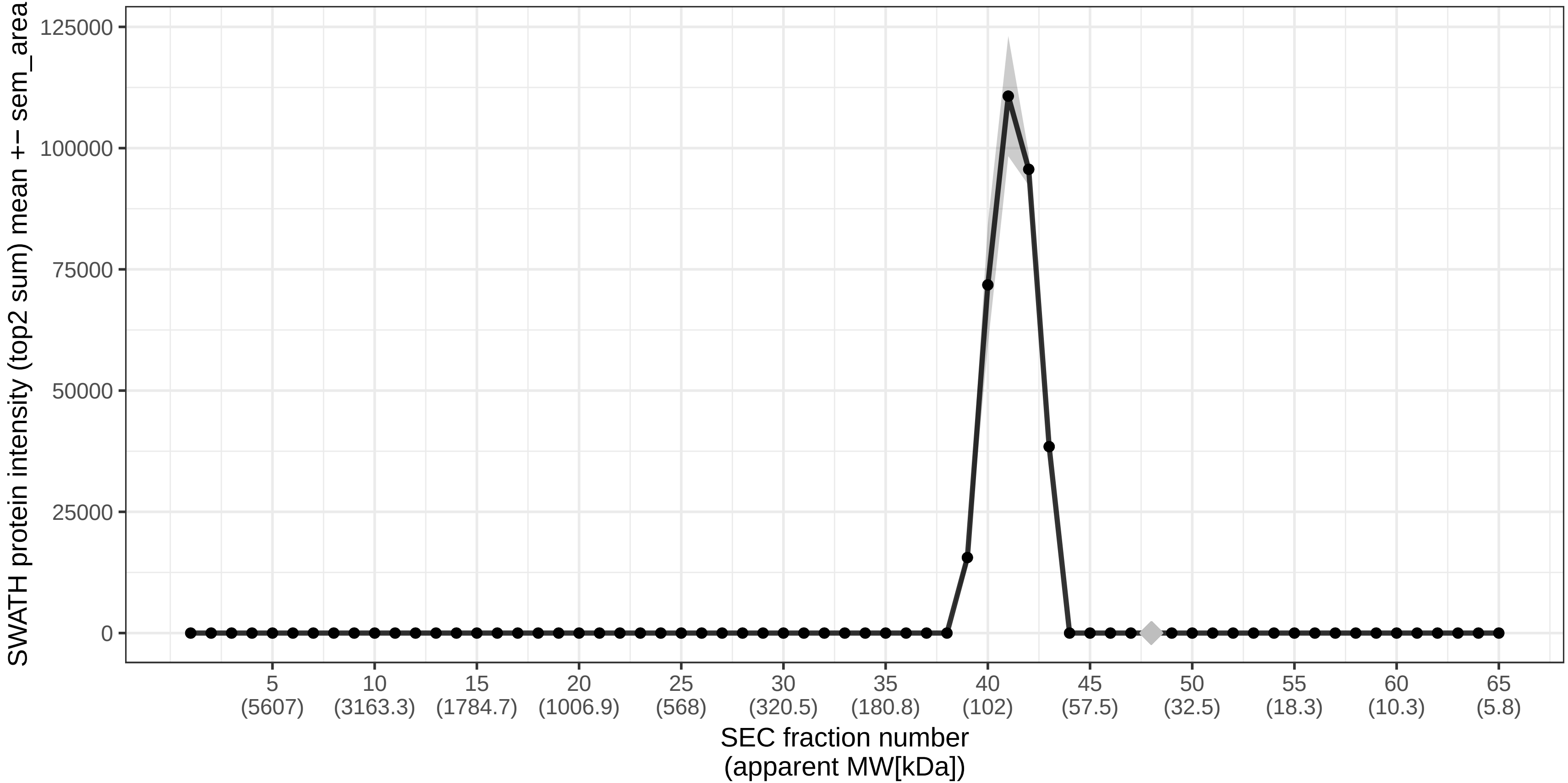

Supplement: Data S1. SEC-SWATH-MS Protein Chromatograms, Related to Figure 1 [file mmc6.zip › SECchrom_Q96G28_CFA36_HUMAN_CFAP36_CCDC104_UNQ163_PRO189.pdf]
